# 18F-Fluorodeoxyglucose (18F-FDG) PET/CT Appearance of Baastrup’s Disease and Its Multimodality Correlation

**DOI:** 10.7759/cureus.64093

**Published:** 2024-07-08

**Authors:** Ali Z Piracha, Pokhraj P Suthar, Sumeet Virmani

**Affiliations:** 1 Department of Diagnostic Radiology and Nuclear Medicine, Rush Medical College, Chicago, USA; 2 Department of Diagnostic Radiology and Nuclear Medicine, Rush University Medical Center, Chicago, USA

**Keywords:** 18f-fdg pet/ct, multimodality correlation, ct, mri, baastrup's disease

## Abstract

Baastrup's disease (BD), commonly known as "kissing spine syndrome," presents a significant cause of lower back pain, predominantly affecting the lumbar region. Diagnosis is often challenging due to its symptomatology and radiographic presentation. Herein, we present a case series demonstrating the utility of 18F-fluorodeoxyglucose positron emission tomography/computed tomography (18F-FDG PET/CT) in diagnosing BD accurately, particularly in oncologic settings where it may mimic metastatic lesions. Through a series of cases, we demonstrate the distinctive imaging features of BD on 18F-FDG PET/CT and its differentiation from malignancies. In addition, we emphasize the importance of clinical awareness and proper correlation with CT or MRI to avoid misinterpretation. Furthermore, we discuss the pathophysiology, clinical presentation, and diagnostic modalities of BD, highlighting its underdiagnosis and potential to mimic metastasis on imaging. By enhancing recognition of BD's appearance on 18F-FDG PET/CT, this study aims to prevent misdiagnoses, reduce unnecessary investigations, and ultimately improve patient care in oncologic practice.

## Introduction

Baastrup's disease (BD), also known as "kissing spine syndrome," is a common cause of lower or lumbar back pain [1]. This condition arises from the chronic contact of adjacent vertebral spinous processes. Typically, BD is identified through imaging techniques, such as X-ray, computed tomography (CT), or magnetic resonance imaging (MRI) [2]. However, its diagnosis can be challenging due to its symptomatology and radiographic appearance. BD frequently affects the elderly and individuals with degenerative spine conditions. Repetitive stress and motion in the lumbar region lead to inflammation and hypertrophy of the interspinous ligaments, causing the vertebrae to come into painful contact. Patients often report pain exacerbation with extension movements and relief with flexion, which can help differentiate BD from other causes of back pain. In this case series, we examine the role of 18F-fluorodeoxyglucose positron emission tomography/computed tomography (18F-FDG PET/CT) in accurately diagnosing BD. This advanced imaging modality, which combines metabolic and anatomical information, is valuable in oncologic evaluations but is underrecognized for identifying benign conditions like BD. Notably, BD can mimic metastatic lesions on 18F-FDG PET/CT scans due to the increased metabolic activity associated with inflammation [3]. Our objective is twofold: to highlight the diagnostic potential of 18F-FDG PET/CT in distinguishing BD from malignant processes, thereby preventing misdiagnoses in oncologic patients, and to raise awareness among clinicians and radiologists about this frequently misdiagnosed condition. Given the routine use of 18F-FDG PET/CT in cancer staging, recognizing the imaging characteristics of BD is crucial to avoid diagnostic errors. By presenting detailed cases, we demonstrate how 18F-FDG PET/CT can be utilized confidently to diagnose BD. We aim to contribute to improved diagnostic accuracy and patient management, especially in oncologic care where distinguishing between benign and malignant lesions is essential.

## Case presentation

Case 1

**Figure 1:**
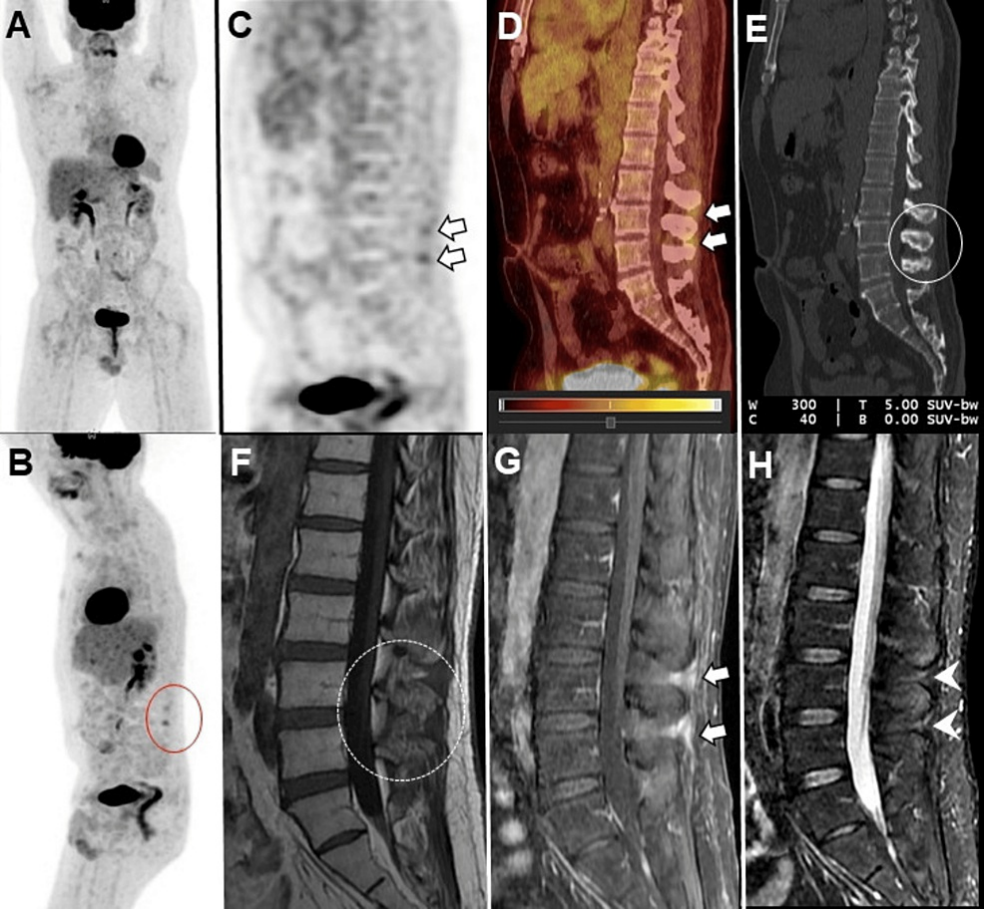
Restaging 18F-FDG PET/CT images: maximum-intensity projection in the frontal (A) and (B) lateral projection, sagittal (C) PET, (E) CT, and (D) fused PET/CT images. No FDG avid nodal disease in the neck, chest, abdomen or pelvis. Incidentally observed increased uptake in the L3-L4 and L4-L5 interspinous ligaments (white arrows) without CT correlation of a destructive osseous lesion (solid open white circle in E). Sagittal (F) T1, (G) T1 post-contrast, and (H) short tau inversion recovery (STIR) MRI lumbar spine images revealed edema (arrowheads in H) and enhancement (solid white arrows in G) in the L3-L4 and L4-L5 interspinous regions, correlating with FDG uptake on 18F-FDG PET/CT without underlying marrow signal abnormality (dashed circle in F).

A 51-year-old male with a history of large B-cell lymphoma underwent treatment with dose-adjusted rituximab, etoposide, prednisolone, vincristine, cyclophosphamide, doxorubicin (DA-R-EPOCH) therapy. Following the completion of this regimen, an 18F-FDG PET/CT scan was performed to assess the therapeutic response. The scan demonstrated a resolution of the previously noted hypermetabolic lymph nodes, indicating a positive response to the treatment. However, the scan also revealed an incidental finding of increased FDG uptake in the L3-L4 and L4-L5 interspinous ligament areas. Notably, there was no corresponding evidence of destructive osseous lesions on the CT component of the PET/CT scan, which could have suggested a more concerning pathology. To further evaluate these findings, an MRI of the lumbar spine was conducted. The MRI confirmed the presence of edema and enhancement in the L3-L4 and L4-L5 interspinous regions, aligning with the areas of increased FDG uptake observed on the PET/CT scan (Figure 1). These imaging characteristics were consistent with BD, a benign condition known for causing back pain due to the close approximation of adjacent spinous processes.

Case 2

**Figure 2:**
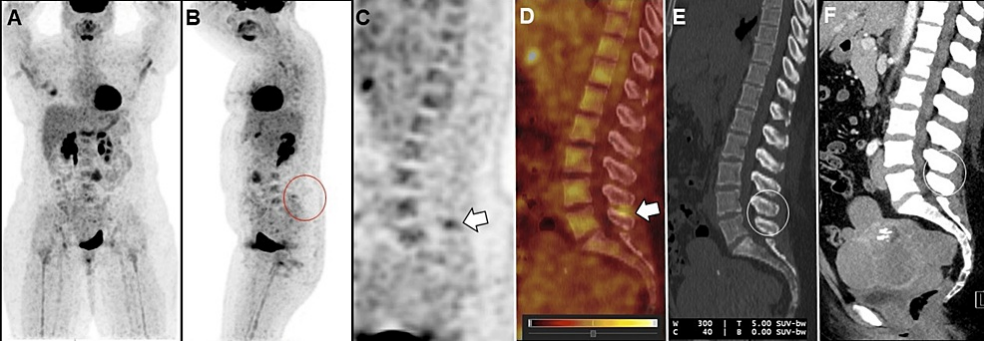
Initial staging 18F-FDG PET/CT images: maximum-intensity projection in the frontal (A) and (B) lateral projection, sagittal (C) PET, (E) CT, and (D) fused PET/CT images. Hypermetabolic right breast mass consistent with known breast cancer. Furthermore, increased uptake in the L4-L5 interspinous ligament (white arrows) without CT correlation of a destructive osseous lesion (open white circle) consistent with Baastrup's disease (BD). (F) A dedicated contrast CT confirmed the absence of any soft tissue mass (open white circle) or destructive lesion at the L4-L5 level, correlating with the FDG uptake on PET/CT. Incidentally, seen are uterine fibroids.

A 49-year-old female with invasive ductal breast carcinoma on initial staging 18F-FDG PET/CT revealed a hypermetabolic right breast malignancy. Furthermore, the 18F-FDG PET/CT showed focal uptake in the L4-L5 interspinous ligament consistent with BD, with dedicated CT confirming the absence of any soft tissue mass or destructive lesion (Figure 2).

Case 3

**Figure 3:**
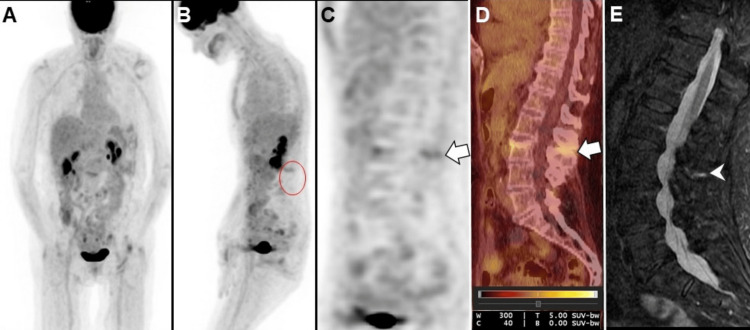
Restaging 18F-FDG PET/CT images: maximum-intensity projection in the frontal (A) and (B) lateral projection, sagittal (C) PET, and (D) fused PET/CT images showed no evidence of FDG avid lymphomatous lesions. However, increased uptake was observed in the L2-L3 interspinous ligament without CT correlation of destructive osseous lesions (white arrows). (E) Short-tau inversion recovery (STIR) sagittal MRI of the lumbar spine revealed edema in the L2-L3 interspinous region (white arrowhead), correlating with FDG uptake on 18F-FDG PET/CT and consistent with Baastrup's disease (BD).

A 78-year-old female with diffuse large B-cell lymphoma in remission underwent a re-staging 18F-FDG PET/CT, with no evidence of FDG avid lymphomatous lesions (Deauville score: 1). However, increased uptake was observed in the L2-L3 interspinous ligament correlating to edema and enhancement in the L2-L3 interspinous region on lumbar spine MRI consistent with BD (Figure 3).

## Discussion

BD is thoroughly documented as a significant source of low back pain, with certain studies indicating a frequency reaching up to 81% among individuals over 80 years of age, although the precise prevalence remains undetermined [4,5].

BD, commonly referred to as "kissing spine disease," clinically presents as chronic low back pain from the close approximation of adjacent spinous processes, predominantly affecting the L4-L5 levels. This proximity often leads to reactive sclerosis, hypertrophy, eburnation, and the development of interspinal bursae due to chronic inflammation resulting from repetitive friction [6]. While prevalent in older patients, it can occasionally be seen in younger populations, particularly gymnasts, due to repetitive flexion and extension [6]. Additional causative factors encompass spinal trauma, poor posture, obesity, and spondylitis [4].

Patients usually present with dull lumbar pain without lateral radiation, alleviated by flexion and exacerbated by extension, along with tenderness upon palpating the involved spinous processes. Diagnosis combines radiological and clinical assessments, with lateral X-rays revealing "kissing spines" and possible sclerosis. CT highlights bony degenerative changes, while the advantage of MRI lies in its lack of radiation exposure and its ability to provide excellent contrast for spinal soft tissues compared to CT [7]. In certain instances of BD, the development of a posterior epidural cyst can occur, adding to the factors contributing to an epidural cyst [8]. The PET/CT scan, conducted to stage a malignant tumor, revealed a distinct 18F-FDG-avid pattern that was essential for precise diagnosis [9]. Frequently underdiagnosed, BD can mimic metastasis on 18F-FDG PET/CT, emphasizing the importance of correlation with CT or MRI. FDG uptake may signify highly vascularized granulation tissue/inflammation at the interspinous ligament-spinous process junction. To avoid misinterpretation, scrutinizing sagittal reconstructions is crucial, especially when involvement is confined to spinous processes, a rarity in malignancy.

## Conclusions

BD often resembles metastatic lesions on 18F-FDG PET/CT, posing diagnostic challenges. Recognizing BD's appearance on 18F-FDG PET/CT is essential to avoid misdiagnosis and unnecessary workup, ultimately improving patient care. BD, or "kissing spine syndrome," is a common cause of lower back pain, particularly in the lumbar region, and its diagnosis is often difficult due to its symptomatology and radiographic presentation. This case series highlights the utility of 18F-FDG PET/CT in accurately diagnosing BD, especially in oncologic settings where it may mimic metastatic lesions. By demonstrating BD's distinctive imaging features and emphasizing the importance of clinical awareness and proper correlation with CT or MRI, we aim to prevent misdiagnoses, reduce unnecessary investigations, and improve patient care in oncologic practice.
